# Preventive Leptin Administration Protects Against Sepsis Through Improving Hypotension, Tachycardia, Oxidative Stress Burst, Multiple Organ Dysfunction, and Increasing Survival

**DOI:** 10.3389/fphys.2018.01800

**Published:** 2018-12-12

**Authors:** Alejandro Vallejos, Pedro Olivares, Diego Varela, Cesar Echeverria, Claudio Cabello-Verrugio, Claudio Pérez-Leighton, Felipe Simon

**Affiliations:** ^1^Facultad de Ciencias de la Vida, Universidad Andrés Bello, Santiago, Chile; ^2^Millennium Institute on Immunology and Immunotherapy, Santiago, Chile; ^3^Programa de Fisiología y Biofísica, Instituto de Ciencias Biomédicas, Facultad de Medicina, Universidad de Chile, Santiago, Chile; ^4^Millennium Nucleus of Ion Channels-Associated Diseases, Universidad de Chile, Santiago, Chile; ^5^Facultad de Medicina, Universidad de Atacama, Copiapo, Chile; ^6^Facultad de Ingeniería, Ciencia y Tecnología, Universidad Bernardo O’Higgins, Santiago, Chile; ^7^Departamento de Fisiología, Facultad de Ciencias Biológicas, Pontificia Universidad Católica de Chile, Santiago, Chile

**Keywords:** sepsis, hypotension, MODS, leptin, endotoxemia, survival

## Abstract

Sepsis syndrome is the most important cause of mortality in critically ill patients admitted to intensive care units (ICUs). However, current therapies for its prevention and treatment are still unsatisfactory, and the mortality rate is still high. Non-septic ICU patients are vulnerable to acquire sepsis syndrome. Thus, a preventive treatment for this population is needed. During sepsis syndrome and endotoxemia, severe hypotension, tachycardia, oxidative and immune response increase, multiple organ dysfunction syndrome (MODS) and decreased survival are observed. Leptin administration protects against negative effects of sepsis syndrome and endotoxemia. Furthermore, it is has been reported that leptin elevates blood pressure mediated by sympathetic nervous system activation. However, whether leptin administration before sepsis induction mediates its protective effects during sepsis through blood pressure regulation is not known. Therefore, we investigated whether pre-treatment of leptin improves blood pressure and MODS, resulting in survival increase during endotoxemia. The results showed that leptin administration before endotoxemia induction reduced both the hypotension and tachycardia characteristically observed during endotoxemia. Notably, this protective effect was observed early and late in the course of endotoxemia. Endotoxemia-induced MODS decreased in leptin-treated rats, which was reflected in normal values for liver and kidney function, inhibition of muscle mass wasting and maintenance of glycemia. Furthermore, leptin pre-treatment decreased the oxidative stress burst in blood and blunted the increased pro-inflammatory cytokines TNF-α, IL-1β, and IL-6 observed during endotoxemia. Remarkably, according to the leptin-induced increase in survival, leptin pre-administration decreased the risk for death associated with sepsis syndrome at early and late times after endotoxemia induction. These results show a potential preventive therapy against sepsis syndrome and endotoxemia in vulnerable patients, based in the beneficial actions of leptin.

## Introduction

Sepsis is the most important cause of mortality in critically ill patients admitted to intensive care units (ICUs) (Riedemann et al., 2003; Zarbock et al., 2014). Sepsis is characterized by the overactivation of the immune system, which involves the secretion of pro-inflammatory cytokines, such as tumor necrosis factor-α (TNF-α), interleukin 1β (IL-1β), IL-6, and the generation of oxidative stress (Riedemann et al., 2003; Pinsky, 2004; Simon and Fernández, 2009; Becerra et al., 2011). Sepsis syndrome is frequently supported by endotoxemia, which is the accumulation of large amounts of Gram-negative bacterial endotoxin, such as lipopolysaccharide (LPS), in the bloodstream (Karima et al., 1999; Riedemann et al., 2003; Pinsky, 2004). A number of basic and clinical studies have addressed sepsis syndrome and endotoxemia; however, current therapies used to treat it and its sequelae are unsatisfactory, and the mortality rate remains high (Rivers and Ahrens, 2008; Winters et al., 2010). Interestingly, the non-septic ICU population and the severely sick patients, are especially susceptible to the sepsis syndrome. Thus, preventive therapies are fundamentally significant for those patient populations.

During sepsis syndrome and endotoxemia, severely decreased blood pressure is observed (Angus et al., 2001; Brun-Buisson et al., 2004; Pinsky, 2004). Because hypotension is a main feature of sepsis syndrome, a well-accepted strategy for sepsis treatment is to maintain stable blood pressure through the incorporation of fluid resuscitation and vasoconstrictor administration (De Backer et al., 2002; Bateman et al., 2003; Vallet, 2003; Hollenberg et al., 2004; Sakr et al., 2004; Trzeciak et al., 2007; Dellinger et al., 2013). This therapeutic strategy has often failed to normalize blood pressure (De Backer et al., 2002; Bateman et al., 2003; Vallet, 2003; Hollenberg et al., 2004; Sakr et al., 2004; Trzeciak et al., 2007; Dellinger et al., 2013).

Sepsis syndrome and endotoxemia are also characterized by extensive organ dysfunction (Trzeciak et al., 2007; Dellinger et al., 2013). Sepsis syndrome that is complicated by organ dysfunction is defined as severe sepsis, and multiple organ dysfunction syndrome (MODS) is a terminal stage of sepsis syndrome, characterized by altered function of several organs (Baue, 2003; Englert and Fink, 2005; Ziesmann and Marshall, 2018).

Many studies have suggested that sepsis-induced hypotension produces organ hypoperfusion, which affects the normal oxygen and glucose supplies and decreases the clearance of carbon dioxide and waste molecules (Marshall, 2001; Ziesmann and Marshall, 2018). Thus, hypotension-induced hypoperfusion generates appropriate conditions to induce MODS, severely elevating the mortality rate of sepsis (Englert and Fink, 2005; Vincent et al., 2007).

It is has been reported that serum leptin levels are involved in the development and outcomes of sepsis syndrome and endotoxemia (Tzanela et al., 2006; Koch et al., 2010). Leptin is an adipocyte-derived protein generated from the *ob* gene. It is a member of the interleukine-6 family of cytokines. Leptin was originally identified as a key factor in the control of food intake and body weight (Allison and Myers, 2014). However, it is now known that leptin also functions as an immune system modulator (Lord et al., 1998; Fantuzzi and Faggioni, 2000; Faggioni et al., 2001; La Cava and Matarese, 2004; Lago et al., 2008). For this reason, the participation of leptin in sepsis and endotoxemia has received increasing attention during the last decade (Tschop et al., 2010a; Behnes et al., 2012; Chen et al., 2014).

A number of studies have shown that the administration of leptin induces protection during sepsis syndrome and endotoxemia by reducing the immune response and other deleterious characteristics (Dong et al., 2013; Landgraf et al., 2014; Negrin et al., 2017). Lipopolysaccharide-induced acute lung injury is lessened by exogenous leptin administration (Dong et al., 2013; Landgraf et al., 2014), and hyperleptinemia generates resistance to endotoxemia (Madiehe et al., 2003), improving sepsis survival and modulating the immune response (Siegl et al., 2014). In the same line, decreased plasma leptin enhances susceptibility to mortality induced by endotoxemia (Faggioni et al., 2000). In concordance, sensitivity to endotoxin-induced lethality was enhanced in leptin-deficient mice (Faggioni et al., 1999). In contrast, some data have shown no effects or deleterious actions of leptin during sepsis or endotoxemia (Shapiro et al., 2010).

Thus, current evidence supports that plasma leptin exerts protective actions during sepsis syndrome and endotoxemia. It has been reported that chronic leptin administration activates the sympathetic nervous system (SNS), elevating blood pressure mediated by α- and β-adrenergic stimulation (Hall, 2001; Masuo, 2002; da Silva et al., 2009; Kalil and Haynes, 2011). However, if leptin administration improves hypotension during sepsis is not known. Therefore, we focused on investigating whether leptin improved blood pressure and MODS during endotoxemia generated in rats.

Here, we showed for the first time, that leptin administration before sepsis induction reduced both the severely decreased systolic blood pressure and increased heart rate that are characteristically observed during endotoxemia such that these parameters were nearly at control levels. Of note, this protective effect of leptin pretreatment was observed at early and late times after endotoxemia induction.

Importantly, endotoxemia-induced MODS was decreased in leptin-treated rats as these animals showed normal kidney and liver function values, decreased muscle mass wasting, and normal glycemia maintenance. Furthermore, leptin treatment decreased the oxidative stress burst in the blood and blunted the increased pro-inflammatory cytokines TNF-α, IL-1β, and IL-6 observed during endotoxemia. Remarkably, according to the leptin-induced increase in survival, leptin administration decreased the risk of death associated with sepsis syndrome at early times after endotoxemia induction, which extended to later times.

Together, these results show a leptin-based potential preventive therapy against sepsis syndrome and endotoxemia for vulnerable patients.

## Materials and Methods

### Animals, Experimental Groups, and Basic Procedures

Male Sprague–Dawley rats, weighing from 100 to 120 g and approximately 4–5 weeks old were used. This study was carried out in accordance with the recommendations of Commission of Bioethics guidelines, Universidad Andrés Bello. Experimental protocols were approved by the Commission of Bioethics and Biosafety of the Universidad Andrés Bello. Rats were separated into four groups: Group 1: the vehicle-treated/saline-treated group; rats were subjected to IP injections of leptin’s vehicle solution (vehicle-treated rats) and were challenged with sham endotoxemia 3 days (72 h) later (rats injected with saline solution) for 24 h (*N* = 8). Group 2: the leptin-treated/saline-treated group; rats were subjected to IP injections of leptin (1 mg/kg) twice a day. After 3 days (72 h) of leptin administration, rats were challenged with sham endotoxemia (saline solution) for 24 h simultaneously with fourth leptin administration (4^th^ day of leptin treatment) (*N* = 8). Group 3: vehicle-treated/endotoxemic group; rats were subjected to IP injections of leptin’s vehicle solution (vehicle-treated rats), and, 3 days (72 h) later, endotoxemia was produced by IP injection of 20 mg/kg LPS (endotoxemic rats) for 24 h, simultaneously with the second vehicle dose administration (*N* = 8). Group 4: leptin-treated/endotoxemic group; rats were subjected to IP injections of leptin (1 mg/kg) twice a day, and, after 3 days (72 h) of leptin administration, endotoxemia was produced by IP injection of 20 mg/kg LPS (endotoxemic rats) for 24 h simultaneously with the leptin administration (4^th^ day of leptin treatment) (*N* = 8). A schematic presentation of all experimental groups is showed in the Supplementary Figure S1. Daily food intake was determined by subtracting the weight of the food left in the cage from the weight of the food put into the cage. Rats were housed in individual cages. Changes in body weight were also recorded daily. To evaluate the final outcome of survival, a time-course of 72 h was used in all of the experimental groups described above (Figure 7) (see Supplementary Figure S1, 72-h protocol).

### Systolic Blood Pressure and Heart Rate

To confirm the effectiveness of the generation of endotoxemia and detect changes to them as consequences of leptin treatment, systolic blood pressure (P_S_) and instantaneous heart rate (f_H_) were measured in conscious animals after saline or endotoxin treatment with a physiological recording acquisition system and a pressure tail cuff for non-invasive blood pressure recording system for rats (ML125/R), coupled with a MLT125/R pulse transducer (ADInstruments, Castle Hill, NSW, Australia). To perform the recordings of P_S_ and f_H_, animals were conscious and placed in a supine position. Tidal volume (V_T_) and instantaneous respiratory frequency (f_R_) were measured by transiently introducing (1 min) the rat head into a plastic mask connected to a respiratory flow head (MLT1L, ADInstruments) that measured the ventilatory flow (δV/δt), which was converted into V_T_ through a volumetric differential pressure transducer. All transducers were connected to a PowerLab 8/30 (ADInstruments), and physiological variables were instantaneously displayed through Chart software (ADInstruments).

### Histological Studies Through Hematoxylin and Eosin Staining

The kidneys and livers were extracted from each experimental group 24 h after the challenge. Formalin-fixed, paraffin-embedded samples were sectioned and subjected to hematoxylin and eosin staining. Blind analysis of the liver and kidney tissue damage was recorded using a tissue damage score following this grading scale: normal = 0, minimal = 1, mild = 2, moderate = 3, marked = 4, and severe = 5. The analysis criteria included several tissue damage characteristics, such as, for liver: morphological changes, cytoplasmic vacuolation, neutrophil/polymorphonuclear infiltration, and erythrocyte accumulation; and for kidney: enhanced swelling of renal cells, glomerular structure alteration and swelling, vacuolar degeneration, and tubular cell necrosis.

### Plasma Measurements of Oxidative Stress, Pro-inflammatory Cytokines, and Multiple Organ Dysfunction Markers

Twenty-four hours after saline or endotoxin administration, cardiac puncture was performed for blood extraction into lithium heparin-containing tubes. The blood was immediately centrifuged at 4,000 rpm for 10 min at 4°C to separate the plasma, which was then used to measure oxidative stress, TNF-α, IL-1β, IL-6, IL-10, and markers of multiple organ dysfunction (MOD). Plasma reactive oxygen species (ROS) were measured in peripheral blood mononuclear cells (PBMCs) using the ROS-sensitive probe, 2,7-dichlorofluorescin diacetate (DCFH-DA). DCFH-DA is a stable, non-fluorescent and cell-permeable molecule that is hydrolyzed by intracellular esterases into the non-fluorescent 2,7-dichlorofluorescin (DCFH), which is oxidized in the presence of peroxides into the highly fluorescent 2,7-dichlorofluorescein (DCF), which is detected at 488 nm. Reduced glutathione (GSH) levels were measured from 0.2 ml of the plasma fractions mixed with 2.25 ml of 0.1 mol/l K-phosphate buffer (pH 8.0) and 25 μl of Ellman’s reagent (10 mmol/l dithionitrobenzoic acid in methanol). After 1 min, the assay absorbance was measured at 412 nm. TNF-α, IL-1β, IL-6, and IL-10 were measured with an enzyme-linked immunosorbent assay (ELISA), according to the manufacturers’ instructions (R&D Systems, Inc., Minneapolis, MN, United States). To measure markers of MOD, we measured plasma levels of aspartate aminotransferase (AST), alanine aminotransferase (ALT), total bilirubin (TBIL), and gamma-glutamyl transferase (GGT) for the liver; creatinine (CRE) and blood urea nitrogen (BUN) for the kidney; creatine kinase (CK) for muscle mass wasting; and glycemia (GLY) for metabolic function. MOD markers were measured with the Piccolo Xpress Chemistry Analyzer (MetLyte and General Chemistry, 13 panel) (Abaxis, Union City, CA, United States) and the iSTAT System (CG4+ cartridge) (Abbott Laboratories, Lake Bluff, IL, United States), according to the manufacturers’ instructions.

### STAT Phosphorylation Determination

Whole hypothalamus were extracted from rats treated with the vehicle or leptin, lysed in a cold lysis buffer with phosphatase inhibitors, and then proteins were extracted. Supernatants were collected and stored in the same lysis buffer. The protein extract and supernatant were subjected to SDS-PAGE, and the resolved proteins were transferred to a nitrocellulose or PVDF membrane. The blocked membrane was incubated with the primary antibody against the phosphorylated form of STAT3 (p-STAT), washed twice, and incubated with a secondary antibody peroxidase-conjugated IgG antibody. Peroxidase activity was detected through enhanced chemiluminescence (Bio-Rad, Berkeley, CA, United States), and images were acquired using Fotodyne FOTO/Analyst Luminary Workstations Systems (Fotodyne, Inc., Hartland, WI, United States). Then, the membrane was stripped, blocked and incubated with a primary antibody against STAT3 and detected as described above. Protein content was determined using densitometric scanning of immunoreactive bands, and intensity values of p-STAT3 were obtained through the densitometry of individual bands normalized against the total STAT3 used as a loading control.

### Quantitative RT-PCR

QPCR experiments were performed to measure the NF-κB mRNA expression in PBMCs. Total RNA was extracted with Trizol according to the manufacturer’s protocol (Invitrogen, Carlsbad, CA, United States). DNAse I-treated RNA was used for reverse transcription using the Super Script II Kit (Invitrogen, Carlsbad, CA, United States). Equal amounts of RNA were used as templates in each reaction. QPCR was performed using the SYBR Green PCR Master Mix (AB Applied Biosystems, Foster City, CA, United States). Assays were run using a Rotor-gene system (Corbet Research) instrument. Data are presented as relative mRNA levels of the gene of interest normalized to relative levels of 28S mRNA.

### Reagents

Leptin and LPS from *E. coli* (serotype 0127:B8) were purchased from Sigma-Aldrich (St. Louis, MO, United States). Buffers and salts were purchased from Merck Biosciences (Darmstadt, Germany). DCFH-DA was purchased from Invitrogen Life Technologies (Carlsbad, CA, United States). Total STAT3 (1:5,000 dilution was used, Cat. N#4904) and p-STAT3 (1:1,000 dilution was used, Cat. N#9145) were purchased from Cell Signaling (Beverly, MA, United States).

### Statistical Analysis

All results are presented as the mean ± SD or mean ± 95% confidence interval (CI) for the relative risk. Differences were considered significant at *p* < 0.05. Significant differences in systolic blood pressure recording experiments were assessed by one-way ANOVA followed by Dunnett’s post-test to compare them with basal recordings and by two-way ANOVA followed by the Bonferroni post-test to compare the vehicle-treated/endotoxemic group with the leptin-treated/endotoxemic group recordings (see the figure legends for detailed explanations). STAT3 phosphorylation was assessed by Student’s *t*-test (Mann-Whitney). Plasma measurements were performed by one-way ANOVA (Kruskal–Wallis) followed by Dunn’s post-test. Contingency analyses with Fisher’s exact test were used to assess the relative risk of death. Kaplan–Meier curves, the log-rank and Gehan–Breslow–Wilcoxon tests were used to determine survival rates.

## Results

### Leptin Administration in Rats Subjected to Endotoxemia Improves Systolic Blood Pressure and Heart Rate Variability

To investigate whether leptin treatment exerts protective effects on the systolic blood pressure (P_S_) and heart rate (f_H_) during endotoxemia, we performed experiments in leptin-treated rats subjected to endotoxemia, in which P_S_ and f_H_ were recorded.

To ensure that leptin administration generated physiological effects, food intake was measured daily for 4 days. Leptin treatment significantly decreased food intake starting on the second day of administration compared to vehicle injection, indicating that leptin reached the bloodstream and induced its canonical actions (Supplementary Figure S2A). In addition to this, to verify that leptin injection was able to reach circulatory system, the plasma leptin was measured for all of the experimental conditions. The results showed that the plasma leptin increased when leptin was injected. No significant changes in the leptin levels were detected when endotoxemia was induced (Supplementary Figure S3).

Considering that leptin acts on the central nervous system (CNS) at the hypothalamus, STAT3 phosphorylation in hypothalamic neurons was determined to demonstrate that leptin reached CNS neurons. After 24 h of leptin administration, significant STAT3 phosphorylation was observed in the hypothalamic samples extracted from leptin-treated rats (Supplementary Figures S2B,C), which was concordant with the decrease in food consumption at 48 h after leptin injection. These results demonstrated that our model of leptin administration generated a hyperleptinemic condition when endotoxemia was produced (at 72 h in Supplementary Figure S2). Additionally, taking into account that the long-term consequence of the decrease in food consumption was weight loss, we measured the rats’ weights daily to detect changes. Leptin-treated rats showed significant decrease in body weight 5 days after leptin administration compared to vehicle-treated rats (Supplementary Figure S2D), demonstrating that leptin administration was able to produce its physiological effects.

Endotoxin administration was effective in inducing cardiovascular changes that were concordant with the accepted criteria for the diagnosis of severe sepsis in humans, which is characterized by hypotension, tachycardia and tachypnea (Levy et al., 2003a,b). To demonstrate this, we recorded the P_S_, f_H_, tidal volume (V_T_), respiratory frequency (f_R_) and minute ventilatory volume (V_E_: V_T_ × f_R_) of the rats 80 min after IP saline or endotoxin challenge. Rats that were subjected to endotoxemia exhibited a significant decrease in systolic blood pressure (hypotension), tachycardia and tachypnea compared to rats that were injected with saline solution, indicating that the LPS treatment generated hemodynamic alterations concordant with severe sepsis (Supplementary Table S1). To evaluate whether leptin administration exerted effects on hemodynamic function, we recorded P_S_, f_H_, V_T_, f_R_, and V_E_ 96 h after leptin administration, to evaluate the generation of hypotension, tachycardia, or tachypnea. Vehicle-treated and leptin-treated rats did not show any signs of hypotension, tachycardia, or tachypnea when treated with leptin (Supplementary Table S2).

Once our model of leptin treatment and endotoxemia in rats was established, we continued to investigate leptin administration’s role in P_S_ and f_H_ levels during endotoxemia. To that end, we measured these variables for 80 min immediately after endotoxemia induction in rats treated with vehicle or leptin.

**FIGURE 1:**
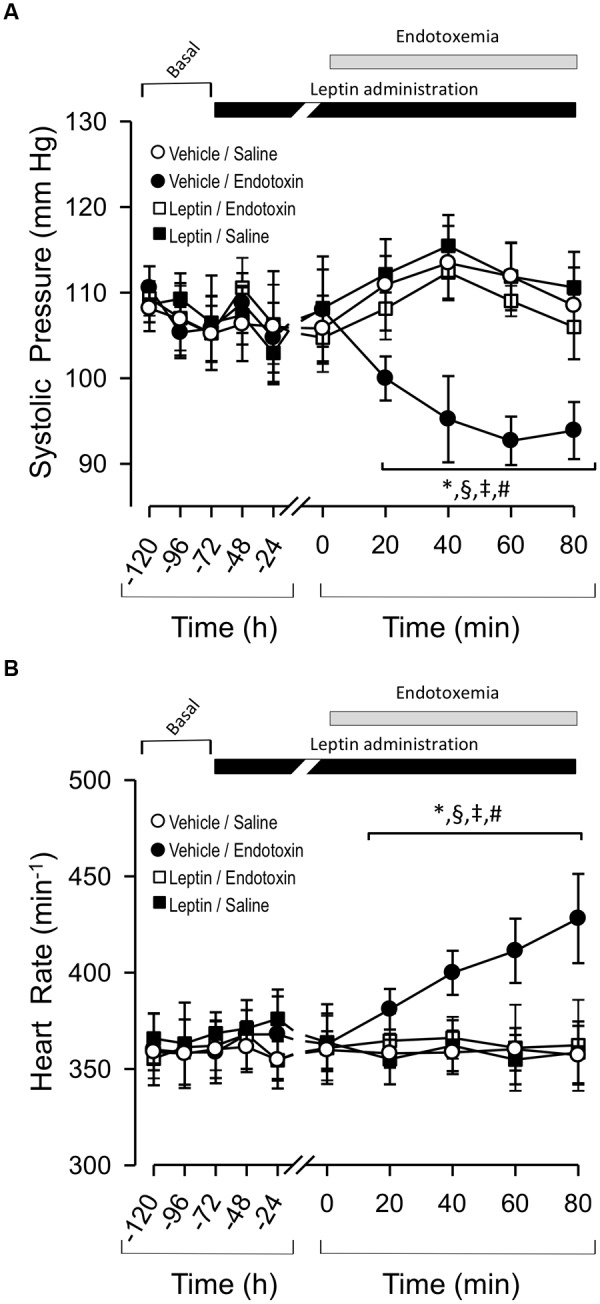
Leptin administration improves hypotension and tachycardia at early time course in endotoxemic rats. Systolic blood pressure (P_S_) **(A)** and heart rate (f_H_) **(B)** variables in vehicle-treated/saline-treated rats (open circles, *N* = 8), leptin-treated/saline-treated rats (open squares, *N* = 8), vehicle-treated/endotoxemic rats (closed circles, *N* = 8), and leptin-treated/endotoxemic rats (closed squares, *N* = 8). Leptin was administrated by IP injection (1 mg/kg) twice a day for 4 days. After 3 days of leptin administration, endotoxemia was produced by IP injection of 20 mg/kg LPS. Animals were recorded 2 days in basal condition (basal) and then 3 days with leptin or vehicle administration (leptin administration). Next, animals were challenged with LPS or saline solution for 80 min. Values are expressed as the mean ± SD. ^∗^*p* < 0.05, vehicle-treated/endotoxemic group compared to basal, assessed by ANOVA and Dunnett’s post-test. ^§^*p* < 0.05, vehicle-treated/endotoxemic group compared with leptin-treated/ endotoxemic group, assessed by two-way ANOVA and the Bonferroni post-test. ^‡^*p* < 0.05, vehicle-treated/endotoxemic group compared with vehicle-treated/saline-treated group, assessed by two-way ANOVA and the Bonferroni post-test. ^#^*p* < 0.05, vehicle-treated/endotoxemic group compared with leptin-treated/saline-treated group, assessed by two-way ANOVA and the Bonferroni post-test. *N* = 8.

Rats injected twice daily IP with leptin’s vehicle solution (vehicle-treated rats) were challenged with sham endotoxemia (rats injected with saline solution) 3 days later. This vehicle-treated/saline-treated group did not show changes in P_S_ or f_H_ compared to the leptin-treated rats that were injected with saline solution (leptin-treated/saline-treated group) (Figures 1A,B, respectively). However, vehicle-treated rats subjected to endotoxemia (vehicle-treated/endotoxemic group) showed an acute and significant decrease in P_S_ (Figure 1A) and increase in f_H_ (Figure 1B) 20 and 40 min after the endotoxemia challenge, respectively. These changes are concordant with the induction of endotoxemia. Notably, leptin-treated rats that were subjected to endotoxemia (leptin-treated/endotoxemic group) were resistant to changes in P_S_ and f_H_ levels and did not show values that were different from those of the vehicle-treated/saline-treated rats (Figures 1A,B, respectively). These results suggest that the administration of exogenous leptin generates an acute cardiovascular protective action to maintain normal P_S_ and f_H_ during endotoxemia.

**FIGURE 2:**
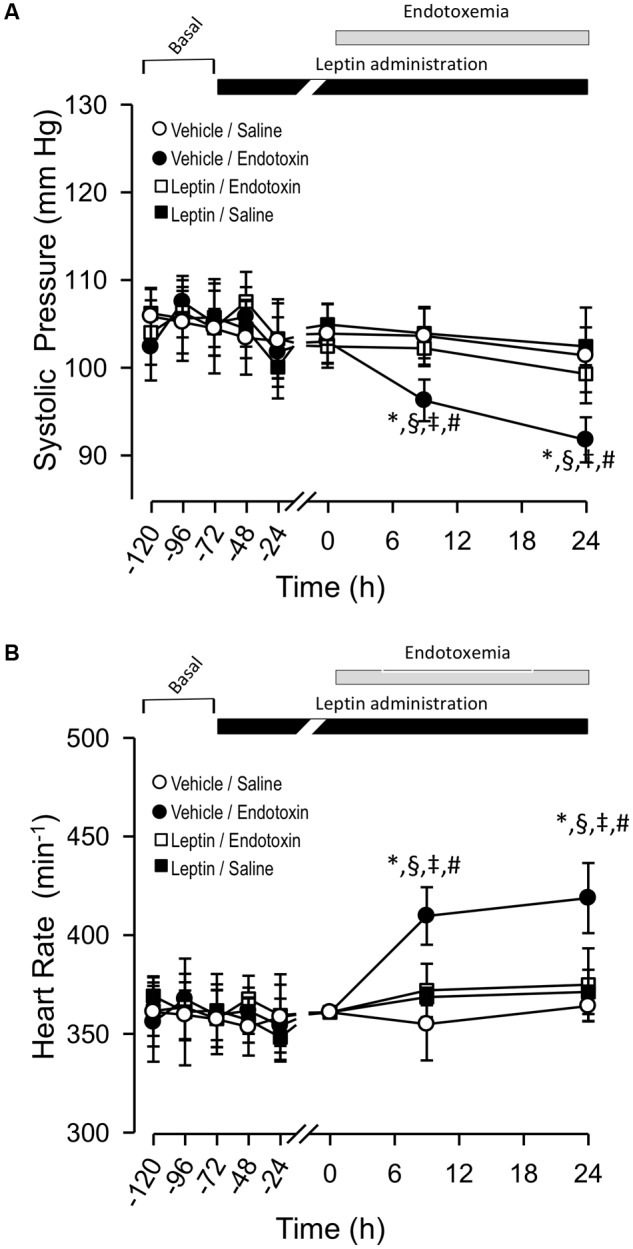
Leptin administration improves hypotension and tachycardia at late time course in endotoxemic rats. Systolic blood pressure (P_S_) **(A)** and heart rate (f_H_) **(B)** variables in vehicle-treated/saline-treated rats (open circles, *N* = 8), leptin-treated/saline-treated rats (open squares, *N* = 8), vehicle-treated/endotoxemic rats (closed circles, *N* = 8), and leptin-treated/endotoxemic rats (closed squares, *N* = 8). Leptin was administrated by IP injection (1 mg/kg) twice a day for 4 days. After 3 days of leptin administration, endotoxemia was produced by IP injection of 20 mg/kg LPS. Animals were recorded 2 days in basal condition (basal) and then 3 days with leptin or vehicle administration (leptin administration). Next, animals were challenged with LPS or saline solution for 24 h. Values are expressed as the mean ± SD. ^∗^*p* < 0.05, vehicle-treated/endotoxemic group compared to basal, assessed by ANOVA and Dunnett’s post-test. ^§^*p* < 0.05, vehicle-treated/endotoxemic group compared with leptin-treated/endotoxemic group, assessed by two-way ANOVA and the Bonferroni post-test. ^‡^*p* < 0.05, vehicle-treated/endotoxemic group compared with vehicle-treated/saline-treated group, assessed by two-way ANOVA and the Bonferroni post-test. ^#^*p* < 0.05, vehicle-treated/endotoxemic group compared with leptin-treated/saline-treated group, assessed by two-way ANOVA and the Bonferroni post-test. *N* = 8.

Next, we determined whether endotoxemia-induced P_S_ and f_H_ variability would also be modulated by leptin treatment with a more extended time-course. Thus, we investigated changes in these variables for up to 24 h after endotoxemia induction. Vehicle-treated/saline-treated rats did not show changes in P_S_ and f_H_ compared to leptin-treated/saline-treated rats (Figures 2A,B, respectively). In contrast, as we expected, the vehicle-treated/endotoxemic group showed a late and significant decrease in P_S_ (Figure 2A) and increase in f_H_ (Figure 2B). Remarkably, in concordance with the results shown in Figure 2, leptin-treated/endotoxemic rats were resistant to changes in P_S_ and f_H_ levels and did not show values that were different from those of the vehicle-treated/saline-treated rats (Figures 2A,B). These results indicate that leptin treatment generates a late cardiovascular protective effect to maintain normal P_S_ and f_H_ during endotoxemia.

Because NO regulates vasorelaxation, which contributes to the regulation of blood pressure, it is plausible that leptin exerts actions that modulate NO biodisposition to maintain blood pressure during sepsis. Thus, we were prompted to determine the NO plasma levels in all of the experimental condition at 24 h after endotoxin challenge. We did not find any changes in the NO levels in any of the experimental groups (data not shown). This result suggests that leptin could exert a sympathoexcitatory action to increase blood pressure by some means other than by modifying NO levels.

### Leptin Administration Protects Endotoxemic Rats Against Multiple Organ Dysfunction Syndrome and Hypoglycemia

A hallmark of severe sepsis, including endotoxemia, is MODS affecting the functions of several organs, such as the liver, kidney, and muscle. Additionally, metabolic function is extremely altered, producing severe hypoglycemia 12 h after the induction of sepsis. To investigate whether leptin administration is beneficial in maintaining normal liver and kidney function during endotoxemia, we performed experiments in leptin-treated rats subjected to endotoxemia, in which serum markers for liver and kidney dysfunction were measured 24 h after endotoxemia induction.

**FIGURE 3:**
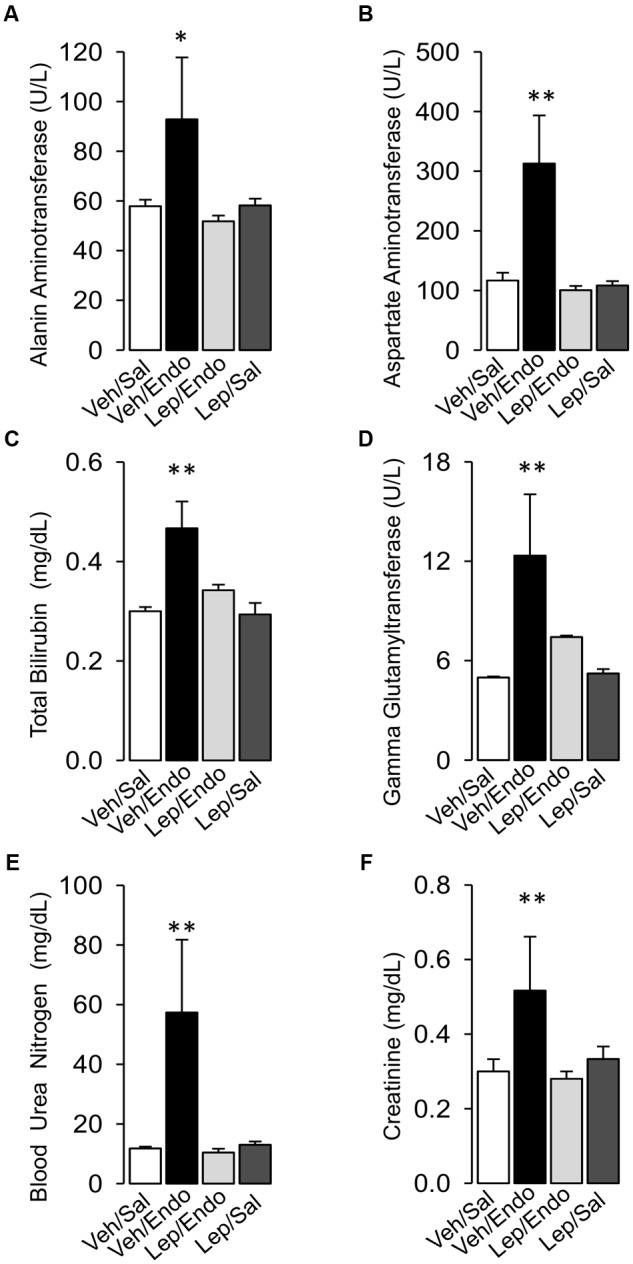
Leptin administration improves MODS in endotoxemic rats. Alanine aminotransferase **(A)**, Aspartate aminotransferase **(B)**, total bilirubin **(C)**, and gamma-glutamyl transferase **(D)** for the liver function and blood urea nitrogen **(E)** and creatinine **(F)** for the kidney function were measured in vehicle-treated/saline-treated rats (open bars, *N* = 8), vehicle-treated/endotoxemic rats (closed bars, *N* = 8), leptin-treated/endotoxemic rats (gray bars, *N* = 8), and leptin-treated/saline-treated rats (dark gray bars, *N* = 8). Values are expressed as the mean ± SD. ^∗^*p* < 0.05; ^∗∗^*p* < 0.01, assessed by one-way ANOVA (Kruskal–Wallis) and Dunn’s post-test. *N* = 8.

Vehicle-treated/saline-treated and leptin-treated/saline-treated rats did not show any changes in the plasma levels of any liver dysfunction marker (AST, ALT, TBIL, or GGT) or kidney dysfunction marker (CRE or BUN) (Figures 3A–F). Saline-treated/endotoxemic rats showed elevated levels of AST, ALT, TBIL and GGT and CRE and BUN, indicating the generation of endotoxemia-induced dysfunction of the liver and kidney, respectively (Figures 3A–F). Interestingly, leptin-treated/endotoxemic rats were resistant to liver and kidney dysfunction as indicated by similar AST, ALT, TBIL, GGT, CRE, and BUN values to those of the vehicle/saline and leptin-treated/saline groups (Figures 3A–F).

**FIGURE 4:**
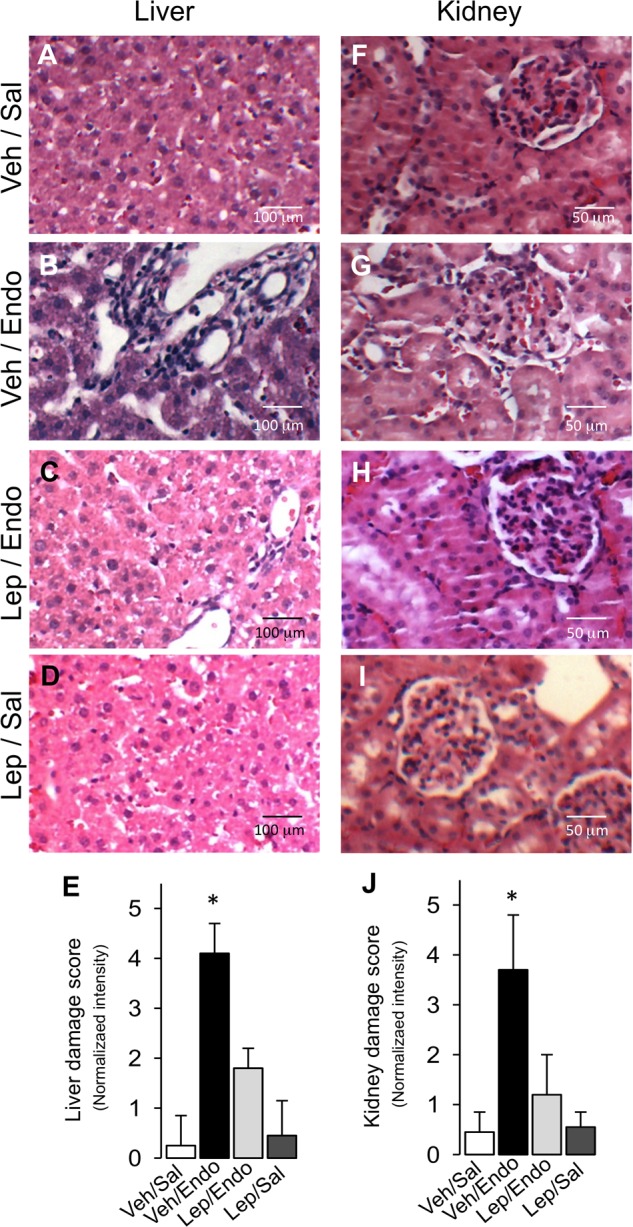
Leptin administration decreases liver and kidney tissue damage in endotoxemic rats. Representative images of Hematoxylin/Eosin-stained liver and kidney sections from vehicle-treated/saline-treated rats **(A,F**), vehicle-treated/endotoxemic rats **(B,G**), leptin-treated/endotoxemic vehicle-treated/endotoxemic rats **(C,H**), and leptin-treated/saline-treated rats **(D,I)**, after 24 h. Images showed in **(A–D,F–I)** were subjected to blind analysis of tissue liver and kidney damage using the following grading scale for tissue damage: normal = 0, minimal = 1, mild = 2, moderate = 3, marked = 4, and severe = 5 **(E,J)**. Values are expressed as the mean ± SD. ^∗^*p* < 0.05, assessed by one-way ANOVA (Kruskal–Wallis) and Dunn’s post-test. *N* = 8.

Histopathological examination corroborated the beneficial effects of leptin treatment during endotoxemia on liver and kidney function. After endotoxemia induction, liver tissue sections showed morphological changes, the appearance of cytoplasmic vacuoles, polymorphonuclear infiltration and erythrocyte accumulation (Figure 4B), compared with the Veh/Sal condition (Figure 4A) and Lep/Sal condition (Figure 4D). Endotoxemia-induced signs of damage were decreased in leptin-treated animals (Figure 4C), as indicated by a blindly scored grading scale for liver damage (Figure 4E). Furthermore, kidney samples showed enhanced swelling of renal cells, glomerular structure alteration and swelling, vacuolar degeneration and tubular cell necrosis in the endotoxemia condition (Figure 4G), compared with the Veh/Sal condition (Figure 4F) and Lep/Sal condition (Figure 4I). All of these detrimental features of renal endotoxemia-induced damage were also decreased by leptin administration (Figure 4H), as indicated by a blindly scored grading scale for kidney damage (Figure 4J).

**FIGURE 5:**
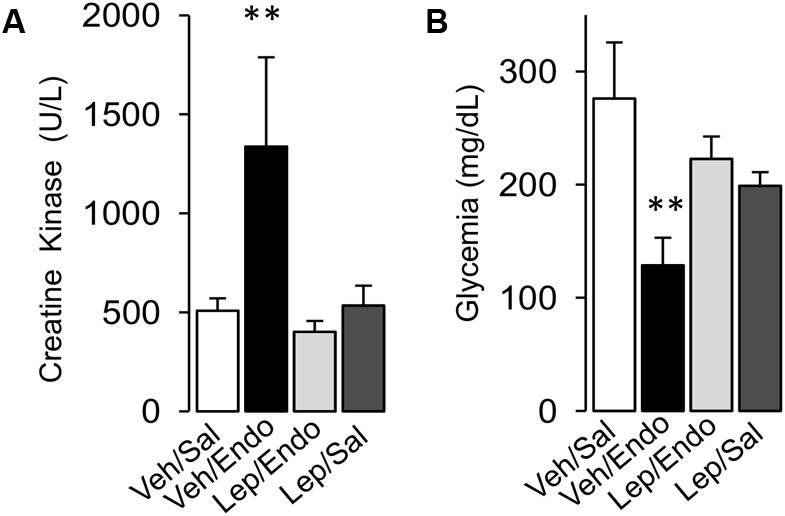
Leptin administration improves mass muscle wasting and hypoglycemia in endotoxemic rats. Creatine kinase **(A)** for muscle mass wasting and glycemia **(B)** for glycemia control were measured in vehicle-treated/saline-treated rats (open bars, *N* = 8), vehicle-treated/endotoxemic rats (closed bars, *N* = 8), leptin-treated/endotoxemic rats (gray bars, *N* = 8), and leptin-treated/saline-treated rats (dark gray bars, *N* = 8). Values are expressed as the mean ± SD. ^∗∗^*p* < 0.01, assessed by one-way ANOVA (Kruskal–Wallis) and Dunn’s post-test. *N* = 8.

Considering that muscle mass is severely compromised during severe sepsis and endotoxemia, we evaluated whether leptin administration protects against muscle mass wasting during endotoxemia. Vehicle-treated/saline-treated and leptin-treated/saline-treated rats did not show any changes in the plasma levels of creatine kinase (CK) (Figure 5A), whereas vehicle-treated/endotoxemic rats showed increased CK serum levels (Figure 5A). However, leptin-treated/endotoxemic rats showed similar CK levels to those observed in the vehicle/saline and leptin-treated/saline groups (Figure 5A), indicating that leptin administration protected endotoxemic rats from muscle mass wasting.

Furthermore, hypoglycemia is observed in severe sepsis, which affects normal organ function. Taking this into account, we investigated whether leptin treatment reduced the hypoglycemia observed in endotoxemia. Plasma glucose levels were not different between vehicle-treated/saline-treated and leptin-treated/saline-treated rats (Figure 5B). Vehicle-treated/endotoxemic rats showed severe hypoglycemia, which was in agreement with previously published data (Maitra et al., 2000; Igaki et al., 2003; Tsai et al., 2011) (Figure 5B). Remarkably, leptin-treated/endotoxemic rats showed similar plasma glucose levels to those of the vehicle-treated/saline-treated and leptin-treated/saline-treated groups (Figure 5B), demonstrating that leptin administration maintained normal blood glucose during endotoxemia.

Taken together, these results indicate that leptin administration exerts a protective effect against MODS by preventing liver and kidney dysfunction and muscle mass wasting during endotoxemia. Additionally, leptin treatment preserves glycemia at normal levels in endotoxemic rats.

### Leptin Administration in Rats Subjected to Endotoxemia Prevents Oxidative Stress and an Increase in Pro-inflammatory Cytokines

Considering that endotoxemia is characterized by an uncontrolled inflammatory response, we investigated whether leptin treatment was able to prevent the increases in oxidative stress and pro-inflammatory cytokine secretion.

**FIGURE 6:**
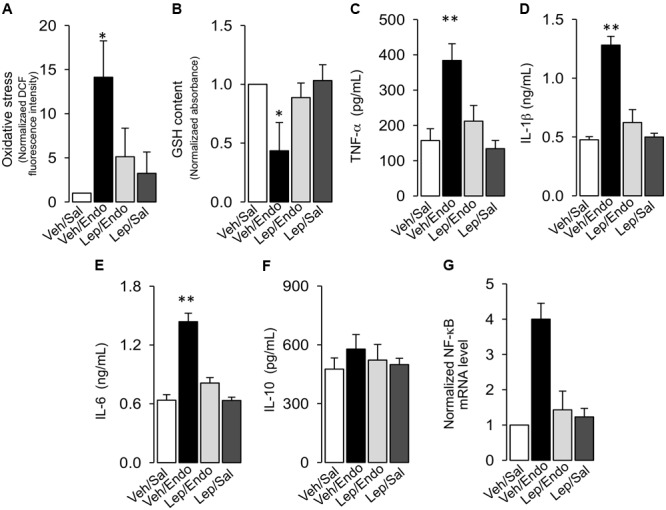
Leptin administration decreases oxidative burst and pro-inflammatory cytokines secretion in endotoxemic rats. Plasma levels of oxidative stress **(A)**, reduced glutathione content **(B)**, TNF-α **(C)**, IL-1β **(D)**, IL-6 **(E)**, IL-10 **(F)**, and NF-κB mRNA levels **(G)** were measured in vehicle-treated/saline-treated rats (open bars, *N* = 8), vehicle-treated/endotoxemic rats (closed bars, *N* = 8), leptin-treated/endotoxemic rats (gray bars, *N* = 8), and leptin-treated/saline-treated rats (dark gray bars, *N* = 8). Oxidative stress was measured as the normalized DCF fluorescence changes and reduced glutathione (GSH) content was measured as the normalized absorbance. Values are expressed as the mean ± SD. ^∗^*p* < 0.05; ^∗∗^*p* < 0.01, assessed by one-way ANOVA (Kruskal–Wallis) and Dunn’s post-test. *N* = 8.

Leptin administration decreased the generation of blood oxidative stress during endotoxemia. Vehicle-treated/saline-treated rats did not show changes in ROS levels in PBMC compared to leptin-treated/saline-treated rats, as measured with the ROS-sensitive dye, dichlorofluorescein (DCF) (Figure 6A). According to endotoxemia progression, the vehicle-treated/endotoxemic group of rats showed a strong increase in ROS, indicating that endotoxemia generated a severe environment of oxidative stress. However, leptin-treated/endotoxemic rats were resistant to the increase in ROS levels in the blood (Figure 6A), with values that were not different compared to than those of the vehicle-treated/saline-treated and leptin-treated/saline-treated group. Similar results were obtained with diacetate dihydroethidium (DHE), a probe that selectively detects superoxide anions (Supplementary Figure S4). According to the results showing that leptin was able to inhibit the endotoxemic oxidative burst, leptin treatment was also efficient in inhibiting the decrease in the levels of the reduced form of glutathione (GSH) in the blood of animals subjected to endotoxemia. Vehicle-treated/saline-treated rats showed no differences in GSH blood levels compared to the leptin-treated/saline-treated rats (Figure 6B), whereas the vehicle-treated/endotoxemic rats showed a decrease in GSH levels, indicating that endotoxemia generated a lower antioxidant condition. However, leptin-treated/endotoxemic rats showed similar GSH levels to those of the vehicle-treated/saline-treated and leptin-treated/saline-treated rats (Figure 6B). Furthermore, there were no differences in TNF-α, IL-1β, IL-6, or IL-10 blood levels between vehicle-treated/saline-treated and leptin-treated/saline-treated rats (Figures 6C–F, respectively). However, vehicle-treated/endotoxemic rats showed strong increased TNF-α, IL-1β, and IL-6 levels, indicating that a severe inflammatory response was evoked by endotoxemia (Figures 6C–F, respectively). IL-10 levels were not changed (Figure 6F). Interestingly, leptin-treated rats subjected to endotoxemia failed to increase the pro-inflammatory cytokine levels. Leptin-treated/endotoxemic rats were resistant to increases in TNF-α, IL-1β, and IL-6 (Figures 6C–E, respectively) and did not show values that were different from those of the vehicle-treated/saline-treated and leptin-treated/saline-treated rats. Considering that NF-κB is linked to cytokine production, we were prompted to measure NF-κB mRNA levels in PBMCs. Our results showed that leptin decreases the endotoxemia-induced increase in the NF-κB mRNA levels (Figure 6G).

### Leptin Administration Decreases the Relative Risk of Death During Endotoxemia

**FIGURE 7:**
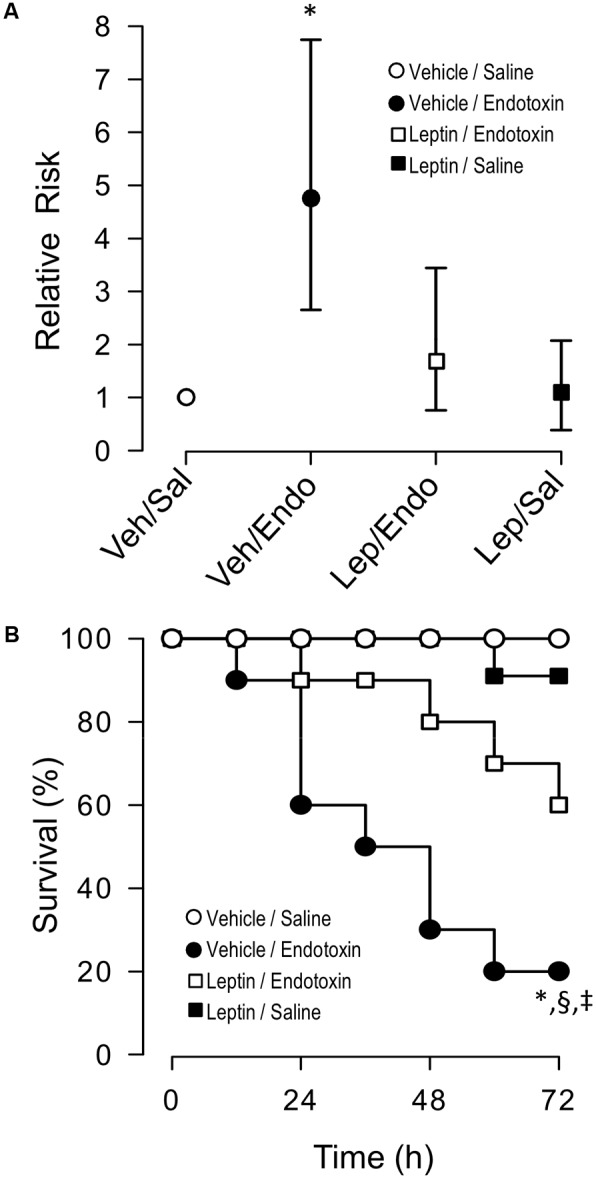
Leptin administration decreases the risk of death and mortality in endotoxemic rats. Contingency analyses **(A)** and survival (Kaplan–Meier) curves **(B)** determined in vehicle-treated/saline-treated rats (open circles, *N* = 8), leptin-treated/saline-treated rats (closed squares, *N* = 8), vehicle-treated/endotoxemic rats (closed circles, *N* = 8), and leptin-treated/endotoxemic rats (open squares, *N* = 8), after 72 h. In **(A)**, values are expressed as the mean ± 95% CI. ^∗^*p* = 0.0298 (Fisher’s exact test) comparing vehicle-treated/endotoxemic rats versus leptin-treated/endotoxemic group. *p* = 0.0044 when comparing vehicle-treated/endotoxemic rats versus vehicle-treated/saline-treated group. *p* = 0.0098 when comparing vehicle-treated/endotoxemic rats versus leptin-treated/saline-treated group. In **(B)**, ^∗^*p* = 0.0023, §*p* = 0.0135, ^‡^*p* = 0.0314 [log-rank (Mantel–Cox) test] when comparing vehicle-treated/endotoxemic rats compared versus vehicle-treated/saline-treated, leptin-treated/saline-treated, and leptin-treated/endotoxemic rats, respectively.

As a next step, a contingency analysis performed by a Fisher’s exact test showed that leptin treatment decreased the risk of death in endotoxemic rats. Concordant with the mortality outcomes of this syndrome, endotoxemic rats (vehicle-treated/endotoxemic rats) showed an elevated risk of death compared to vehicle-treated/saline-treated and leptin-treated/saline-treated rats, 72 h after endotoxemia induction. Interestingly, endotoxemic rats that were treated with leptin (leptin-treated/endotoxemic group) showed a decreased risk of death. The leptin-treated/endotoxemic group was different than the vehicle-treated/endotoxemic rats, whereas the leptin-treated/endotoxemic group showed no differences compared to the vehicle-treated/saline-treated and leptin-treated/saline-treated rats, 72 h after endotoxemia induction (Figure 7A).

In agreement with previously reported results, leptin administration to endotoxemic subjects exerted an improvement in survival. By evaluating death events within a 72 h time frame of endotoxemia, we found that the risk of death significantly decreased in the leptin-treated/endotoxemic group when curves were analyzed using a log-rank test (also called the Mantel–Cox test). A comparison of the survival curves from the vehicle-treated/saline-treated and leptin-treated/saline-treated rats showed no differences between the groups. In contrast, survival curves from the endotoxemic rats (vehicle-treated/endotoxemic rats), showed a significant difference compared to the vehicle-treated/saline-treated and leptin-treated/saline-treated rats, as expected with the high mortality score of sepsis. Remarkably, survival curve analysis of the leptin-treated/endotoxemic group revealed that despite the recorded deaths, leptin administration in endotoxemic animals decreased the risk of death. A comparison of the survival curve of the leptin-treated/endotoxemic group revealed that this condition was not different from those of the vehicle-treated/saline-treated or leptin-treated/saline-treated groups, whereas the comparison with the vehicle-treated/endotoxemic condition showed a significant difference indicating that the relative risk of death was decreased. Giving more weight to deaths at early time points to perform the analysis (Gehan-Breslow-Wilcoxon test) showed that leptin treatment also decreased the risk of death during the early time frame. A comparison of the survival curves with the leptin-treated/endotoxemic group showed that neither the vehicle-treated/saline-treated group nor the leptin-treated/saline-treated group differed from the leptin-treated/endotoxemic group, whereas the vehicle-treated/endotoxemic group showed an increased risk of death (Figure 7B).

## Discussion

Despite some results that have suggested the beneficial effects of leptin administration toward increasing survival during endotoxemia and sepsis syndrome, the role of leptin as a potential preventive therapy against sepsis syndrome and endotoxemia is far from being demonstrated. The results reported here suggest that the protective actions of leptin administration on septic and endotoxemic subjects are based on its ability to maintain blood pressure to preserve normal perfusion to organs and lessening the immune response.

These results show for the first time that administration of exogenous leptin reduced both hypotension and tachycardia observed during endotoxemia, at early and late time points within the course of sepsis, maintaining these parameters to nearly normal levels. Endotoxemia-induced MODS decreased in leptin-treated rats as these rats showed normal values for liver and kidney function, inhibition of muscle mass wasting and maintenance of glycemia. Furthermore, leptin treatment decreased the oxidative stress burst in blood and blunted the increase in pro-inflammatory cytokines TNF-α, IL-1β, and IL-6 observed during endotoxemia. Notably, according to the leptin-induced increase in survival, leptin administration decreased the risk for death associated with endotoxemia at early and late times within the course of sepsis.

Accordingly, leptin is produced and secreted by adipose tissue; obese subjects frequently exhibit resistance to sepsis and endotoxemia. In these cases, it has been proposed that protection is probably mediated through the modulation of pro- and anti-inflammatory cytokine secretion and their subsequent actions (Vachharajani, 2008; Hillenbrand et al., 2010; Pini et al., 2013; Siegl et al., 2014). In agreement with this, it has been reported that leptin produces actions on the CNS to exert neuroendrocrine control of the immune system (Tschop et al., 2010b). It has been shown that leptin increases the inflammatory immune response (Loffreda et al., 1998), which suggests a pathway that contributes to the pathogenesis of obesity. Thus, obese and non-obese hyperleptinemic septic patients can have different outcomes due to the different actions of leptin in these types of patients. In this line, leptin’s actions in non-obese, septic patients has earned increased attention (Tzanela et al., 2006; Koch et al., 2010). Leptin administration to non-obese subjects suffering from sepsis syndrome and endotoxemia improves survival, probably by mediating the activity of the immune response (Madiehe et al., 2003; Dong et al., 2013; Landgraf et al., 2014; Siegl et al., 2014; Negrin et al., 2017). Furthermore, exogenous leptin administration to leptin-deficient mice improved the pulmonary bacterial clearance and survival during pneumococcal pneumonia (Hsu et al., 2007). In agreement with the evidence that shows the beneficial actions of leptin during sepsis and endotoxemia, the alteration of the leptin receptor has been associated with higher mortality in patients with peritonitis (Bracho-Riquelme et al., 2011). According to actions induced by administration of exogenous leptin, decreased leptin levels increase the susceptibility to mortality induced by endotoxemia (Faggioni et al., 2000). Leptin-deficient mice have an impaired resistance to Gram-negative pneumonia (Mancuso et al., 2002) and listeria monocytogenes (Ikejima et al., 2005) and enhanced sensitivity to endotoxin-induced lethality (Faggioni et al., 1999). In contrast, no effect or deleterious actions of leptin have been shown during sepsis or endotoxemia (Shapiro et al., 2010). Further analyses must be performed to elucidate the apparent controversy.

It is interesting that the serum leptin levels are increased during sepsis and endotoxemia (Maruna et al., 2001; Yousef et al., 2010). Several studies have indicated that endotoxin stimulates synthesis of leptin mRNA and protein (Landman et al., 2003). For that reason, serum leptin levels have also been proposed as diagnostic tools, biomarkers and prognostic factors to predict the development of sepsis and its outcomes (Jacobsson et al., 2017).

Our data showing that leptin can improve hypotension and tachycardia during endotoxemia are of major relevance. Because low blood pressure is a key feature of sepsis syndrome, a general strategy for sepsis treatment is the administration of vasoconstrictors and inotropic agents in order to achieve an adequate blood pressure. However, in a number of cases, this approach has often failed to change hypotension to normal levels (Angus et al., 2001; De Backer et al., 2002; Bateman et al., 2003; Vallet, 2003; Brun-Buisson et al., 2004; Hollenberg et al., 2004; Pinsky, 2004; Sakr et al., 2004; Trzeciak et al., 2007; Dellinger et al., 2013). One of the more important consequences of hypotension, particularly refractory hypotension, is insufficient blood perfusion to organs. Signs of hypoperfusion to organs are frequently observed in septic patients, probably as a consequence of decreased blood pressure (Angus et al., 2001; De Backer et al., 2002; Bateman et al., 2003; Vallet, 2003; Brun-Buisson et al., 2004; Hollenberg et al., 2004; Pinsky, 2004; Sakr et al., 2004; Trzeciak et al., 2007; Dellinger et al., 2013).

Leptin is a promising mediator of the SNS. Leptin is able to pass through the blood–brain barrier via a saturated receptor-mediated transport system to reach the CNS (Bouret, 2008). Leptin binds to its receptors in several regions of the CNS, activating neural pathways that increase SNS activity and energy expenditure, and decrease appetite (Rahmouni et al., 2004). Despite leptin-induced SNS activation, the exogenous administration of leptin has little effect on blood pressure, perhaps due to the counterbalancing vasodilator effects of nitric oxide (NO), which is stimulated by leptin (da Silva et al., 2009; Hall et al., 2010).

Because NO regulates vasorelaxation, which contributes to the regulation of blood pressure, it is plausible that leptin exerts actions that modulate NO biodisposition to maintain blood pressure during sepsis. Under physiological conditions, leptin induces endothelium-dependent vasorelaxation by stimulating the production of NO and endothelium-derived hyperpolarizing factor (EDHF). Leptin activates endothelial NO synthase (eNOS) through a mechanism that involves AMP-activated protein kinase (AMPK) and PKB/Akt (Bełtowski, 2012). However, under pathological conditions, the NO-mediated vasodilatory effect of leptin is modified. Resistance to the leptin-induced NO effect is observed in pathological hyperleptinemia, which may result from various mechanisms such as downregulation of leptin receptors, increased levels of circulating C-reactive protein, oxidative stress burst and the overexpression of a suppressor of cytokine signaling (Bełtowski, 2012).

It has been observed that a 6-h leptin infusion as well as long-term administration of leptin induce a significant increase in arterial pressure in rats (Correia et al., 2001a,b). Sustained sympathoexcitation might be required for leptin to increase arterial pressure, for example, through its effect on renal sodium reabsorption. Thus, leptin-induced sympathoactivation appears to be a relevant role for the control of arterial pressure. Further experiments are needed to demonstrate whether this mechanism is also used during sepsis to maintain normal blood pressure.

Chronic administration of leptin is effective in raising blood pressure and heart rate. Increased blood pressure and heart rates induced by leptin are mediated by α- and β-adrenergic stimulation through the SNS (Hall, 2001; Masuo, 2002; da Silva et al., 2009; Kalil and Haynes, 2011). The hypertensive actions of leptin are barely detectable in lean subjects, while obese patients have shown strong leptin-induced increases in blood pressure (da Silva et al., 2009). Supporting this observation, leptin-deficient mice were not hypertensive, despite severe obesity (Mark et al., 1999; Dong et al., 2006), pointing out that hyperleptinemia, not obesity, is the blood pressure regulator factor. Thus, elevated circulating leptin concentrations during sepsis syndrome could maintain blood pressure at normal due to its hypertensive effects, which counteract the hypotension caused by sepsis.

Normal blood pressure ensures adequate perfusion to the organs. This condition guarantees a sufficient delivery of nutrients and waste clearance according to each organ requirements (Marshall, 2001; Ziesmann and Marshall, 2018). However, during severe sepsis and septic shock, hypotension-induced hypoperfusion is observed, which severely alters organ function, leading to MODS (Baue, 2003; Englert and Fink, 2005; Ziesmann and Marshall, 2018). It is well-accepted that pro-inflammatory cytokines, especially TNF-α, generate extensive damage to parenchymatous organs and, subsequently, causes MODS (Tracey et al., 1987; Pinsky, 1994; Douzinas et al., 1997). In line with this observation, our experiments showed that leptin treatment decreased the plasma concentration of TNF-α, IL-1β, and IL-6 in endotoxemic animals. Therefore, we have expected blunted alterations in the levels of biochemical markers of organ damage that are usually changed as a consequence of organ failure (Pinsky, 1994; Yang et al., 2007). In fact, leptin administration decreased MODS in endotoxemic rats, which correlates with the decreased plasma concentrations of TNF-α, IL-1β, and IL-6. In addition to pro-inflammatory cytokines, oxidative stress is a key agent in the generation of MODS during sepsis (Crimi et al., 2006; Prauchner, 2017). In fact, antioxidant molecules have been used to protect against organ failure during sepsis (Sener et al., 2005a,b; Andrades et al., 2011). Leptin administration was efficient in decreasing the oxidative plasma levels in endotoxemic animals, which is associated with the decrease in MODS.

Blood glucose control is lost during sepsis (Levy et al., 2003a,b). Within the first 2 h of sepsis, a hyperglycemic phase is observed, whereas a severe hypoglycemic phase is frequently observed 24 h after sepsis (Maitra et al., 2000; Igaki et al., 2003; Tsai et al., 2011). Thus, sepsis shows an early hyperglycemic phase, followed by a late hypoglycemic phase. Considering that in this study glycemia was measured 24 h after endotoxemia induction, the hypoglycemic stage observed in endotoxemic rats was in according to previously observed (Maitra et al., 2000; Igaki et al., 2003; Tsai et al., 2011). Interestingly, during the hypoglycemic stages of sepsis, glucose uptake in macrophage-rich tissues remains elevated and is independent of changes in glucose and insulin (Maitra et al., 2000). Furthermore, liver function is altered, so delivery of glucose through gluconeogenesis is severely decreased. Here, it was shown that leptin was able to maintain normal glycemic levels by preserving liver function and, possibly, decreasing macrophage actions.

Immune response modulation during sepsis is a major feature of importance in leptin administration. In fact, toxicity exerted by TNF-α is blunted by endogenous serum leptin, which promotes a protective effect during systemic inflammation, such as sepsis (Takahashi et al., 1999). Interestingly, leptin-deficient mice exhibited a reduced capacity to produce the protective cytokine, IFN-γ, which was restored upon leptin administration (Wieland et al., 2005), suggesting that leptin accomplished anti-inflammatory, modulating actions. Furthermore, pro-inflammatory cytokines strongly correlate with blood leptin levels. TNF-α, interleukin and C-reactive protein serum concentrations correlate with leptin levels in septic patients (Maruna et al., 2001; Yousef et al., 2010). Our results are in agreement with those of other groups, showing anti-inflammatory actions during sepsis.

The beneficial actions of leptin during sepsis could be mediated by regulation of the expression of the endotoxin receptor toll-like receptor-4 (TRL-4) and modulation of its activity. Adipocytes and preadipocytes isolated from ob/ob and db/db mice (deficient in leptin and the leptin receptor, respectively) showed increased TLR-4 expression (Batra et al., 2007; Fabersani et al., 2017), which suggests that TLR-4 expression is decreased by leptin and its receptor. Interestingly, in liver pathology, increased sensitivity to endotoxin is independent of the increase in TLR-4 expression or the leptin deficiency, which suggests a complementary mechanism to explain enhanced endotoxin sensitivity in hepatic tissue (Romics et al., 2005). Furthermore, TLR-4 signaling involves NF-κB pathway activation. Adipocytes and preadipocytes from ob/ob and db/db mice show increased NF-κB expression, which indicates that leptin is necessary to inhibit NF-κB pathway (Fabersani et al., 2017). Further experiments are needed to elucidate whether leptin modulates TRL-4 expression or activity to generate protection against sepsis.

Canonical leptin actions are mediated by depolarization of pro-opiomelanocortin (POMC) neurons and the hyperpolarization of the neuropeptide Y and agouti-related protein (NPY/AgRP) neurons, which are dependent on the activation of K_ATP_, BK and TRPC channels, promoting changes in neuron excitability (Elias et al., 1999; Xu et al., 2005). With respect to the beneficial effects of leptin administration during sepsis, it is possible to hypothesize that leptin acts on the excitability of the POMC and NPY/AgRP neurons. In fact, leptin signaling in POMC neurons increases neuronal activity through the activation of TRPC channels, whereas it activates PI3K-dependent K_ATP_ channels that inhibit NPY/AgRP neurons (Zhao et al., 2002). Furthermore, leptin regulates ion channels in other cell types. Leptin promotes trafficking of K_ATP_ channels from cytosolic vesicles to the plasma membrane of β-pancreatic cells by stimulating the AMPK (Chen et al., 2013). Leptin is capable of activating the TRPC4 channel, which is expressed in the endothelium. The absence of this channel in TRPC4 KO mice reduces the entry of endothelial Ca^2+^, which impairs the vasorelaxation that is dependent on the endothelium (Wie et al., 2016). The stimulation of cardiac leptin receptors is associated with JAK2/STAT3 signaling and activation of the MAPK and PI3K pathways, which are involved in myocyte hypertrophy and cardioprotection (Gomez-Hurtado et al., 2014). PI3K signaling has been associated with an increase in K^+^ currents and the improvement of electrical remodeling. This has been observed in adult rat ventricular myocytes, where chronic exposure to leptin increases both the expression and function of transient external K^+^ currents through the positive regulation of Kv4.2 and Kv4.3 in a manner that depends on Akt/PKB kinase (Gomez-Hurtado et al., 2014). In a model of cardiac dysfunction, leptin treatment has the beneficial effects of normalizing cardiac function and reducing arrhythmogenesis. The mechanism involved includes a significant increase in Kv4.2 expression, which normalizes the transient outward potassium current and reduces the action potential reduction (Gomez-Hurtado et al., 2017). Thus, leptin could regulate cells in tissues outside CNS by modulating ion channels. However, confirmation of this idea requires further experiments.

Taken together, here we showed a leptin-based potential preventive treatment against sepsis. Preventive leptin administration increases the survival rate of endotoxemia, based on improving low blood pressure, which is associated with decreases in MODS, oxidative burst and pro-inflammatory cytokine secretion. Notably, the risk of death of endotoxemic condition treated with leptin is significantly decreased. Our study is fundamentally significant for both the non-septic ICU population and the severely sick patients, who are especially susceptible to the sepsis syndrome. Considering that the absence of an effective therapy for sepsis syndrome, the evidence presented here could be a step forward in understanding the value of leptin administration as an effective therapeutic alternative, which should be confirmed by studies in which leptin is administered after endotoxemia challenge.

## Author Contributions

AV, PO, DV, CE, CC-V, CP-L, and FS critically revised and edited the manuscript. AV, PO, CE, CP-L, and FS participated in the research design. AV, PO, CE, DV, and FS conducted the experiments and performed the data analyses. AV, PO, CC-V, CP-L, and FS contributed to the figure design. FS wrote the manuscript.

## Conflict of Interest Statement

The authors declare that the research was conducted in the absence of any commercial or financial relationships that could be construed as a potential conflict of interest.
