# Supplementary material for: Preventive Leptin Administration Protects Against Sepsis Through Improving Hypotension, Tachycardia, Oxidative Stress Burst, Multiple Organ Dysfunction, and Increasing Survival
Source: Front Physiol. 2018 Dec 12;9:1800. doi: 10.3389/fphys.2018.01800 (PMC6299116; doi:10.3389/fphys.2018.01800)

Supplementary Information

Preventive leptin administration protects against sepsis through improving hypotension, tachycardia, oxidative stress burst, multiple organ dysfunction and increasing survival.

Alejandro Vallejos[1,2], Pedro Olivares[1], Diego Varela[3,4], Cesar Echeverria[5,6], Claudio Cabello-Verrugio[1,2], Claudio Pérez-Leighton[5], Felipe Simon[1,2,*]

[1]Facultad de Ciencias de la Vida, Universidad Andres Bello, Republica 239, 8370146, Santiago, Chile.
[2]Millennium Institute on Immunology and Immunotherapy, Alameda 340, 8331150, Santiago, Chile.
[3]Programa de Fisiología y Biofísica, Instituto de Ciencias Biomédicas, Facultad de Medicina, Universidad de Chile, Santiago, 8380453, Chile.
[4]Millennium Nucleus of Ion Channels-Associated Diseases (MiNICAD), Universidad de Chile, Chile.
[5]Facultad de Medicina, Universidad de Atacama, Copayapu 485, 1531772, Copiapo, Chile
[6]Facultad de Ingeniería, Ciencia y Tecnología, Universidad Bernardo OHiggins, Viel 1497, 8370993, Santiago, Chile.
[7]Departamento de Fisiología, Facultad de Ciencias Biológicas, Pontificia Universidad Católica de Chile, Alameda 340, 8331150, Santiago, Chile.

Supplementary Material and Methods

Determination of plasma Leptin

The levels of plasma leptin were assessed using Rat Leptin Elisa kit (Abcam®, Cambridge, MA), according to manufacturer's instructions. Briefly, samples were incubated 2 h at RT. After washing, the enzyme-linked polyclonal antibody was added and then, after washing, the substrate solution was added. The enzyme reaction was read at 450 nm.

Determination of ROS by DHE

Plasma reactive oxygen species (ROS) were measured in peripheral blood mononuclear cells (PBMC) using the ROS-sensitive probe, dihidroethidium (DHE). DHE is a stable, non-fluorescent and cell-permeable molecule that is hydrolyzed by intracellular esterases, which is oxidized in the presence of superoxide anion preferentially.

Supplementary Table Legends

Supplementary Table S1. Cardiorespiratory variables in rats that were subjected to IP saline and endotoxin (20 mg/kg) treatment.

Supplementary Table S2. Cardiorespiratory variables in rats that were subjected to IP vehicle and leptin (1 mg/kg twice a day for 6 days) treatment.

Supplementary Table S1. Cardiorespiratory variables in rats that were subjected to IP saline and endotoxin (20 mg/kg) treatment.

**Variable**	**Saline**	**Endotoxin**	***p*-value**
Ps (mm Hg)	111.5 ± 9.6	82 ± 8.7	$p<0.05$
f_H (min^{-1})	362.6 ± 11.7	448 ± 21.3	$p<0.05$
V_T (mL)	1.03 ± 0.9	1.11 ± 0.8	*NS*
f_R (min^{-1})	75.1 ± 12.3	112.4 ± 9.3	$p<0.05$
V_E (mL/min)	77.4 ± 5.6	124.8 ± 6.1	$p<0.05$

Definition of abbreviations: Ps, systolic blood pressure; f_H, heart rate; V_T, tidal volume; f_R: respiratory rate, V_E: minute ventilator volume. *: p-value < 0.05 when comparing saline v/s endotoxin treatment at 80 min post-treatment. *NS*: non-significant.

Supplementary Table S2. Cardiorespiratory variables in rats that were subjected to IP vehicle and leptin (1 mg/kg twice a day for 6 days) treatment.

Variable	Vehicle	Leptin	*p*-value
Ps (mm Hg)	110.8 ± 6.6	118 ± 5.1	*NS*
f_H (min^{-1})	360.1 ± 13.1	359 ± 15.2	*NS*
V_T (mL)	1.05 ± 0.6	1.02 ± 0.7	*NS*
f_R (min^{-1})	78.4 ± 8.2	79.2 ± 9.1	*NS*
V_E (mL/min)	82.3 ± 4.2	80.8 ± 5.7	*NS*

Definition of abbreviations: Ps, systolic blood pressure; f_H, heart rate; V_T, tidal volume; f_R: respiratory rate, V_E: minute ventilator volume. *NS*: non-significant. Comparison was performed as vehicle v/s leptin treatment at 96 h post-treatment.

Supplementary Figure Legends

Supplementary Figure S1. Schematic representation of experimental protocols. Experimental protocols at 24 and 72 h.

Supplementary Figure S2. Leptin administration actions on food intake, STAT3 phosphorylation and body weight change. Food intake was measured in vehicle-treated (open squares, N=8) and leptin-treated (closed circles, N=8) for 144 h (A). Representative images from western blot experiments performed for detection of pSTAT3 and total STAT3 from vehicle-treated and leptin-treated rats (B). Densitometric analyses of the experiments shown in B. p-STAT3 protein levels were normalized against total STAT3 and expressed relative to vehicle-treated condition ($N = 6$) (C). Weight change was measured in vehicle-treated (open squares, N=8) and leptin-treated (closed circles, N=8) for 144 h (D). For (A and D) values are expressed as the mean ± SD. *, $p<0.05$, assessed by two-way ANOVA and the Bonferroni post-test. For (C) values are expressed as the mean ± SD. **, $p<0.01$, assessed by student's t-test (Mann-Whitney).

Supplementary Figure S3. Plasma leptin determinations. Plasma level of leptin was measured in vehicle-treated/saline-treated rats (open bars, N=4), vehicle-treated/endotoxemic rats (closed bars, N=4), leptin-treated/endotoxemic rats (grey bars, N=4), and leptin-treated/saline-treated rats (dark grey bars, N=4). Oxidative stress was measured as the normalized DHE fluorescence changes. Values are expressed as the mean ± SD. *, $p<0.05$, assessed by one-way ANOVA (Kruskal-Wallis) and Dunn's post-test.

Supplementary Figure S4. Leptin administration decreases oxidative burst in endotoxemic rats. Plasma level of oxidative stress was measured in vehicle-treated/saline-treated rats (open bars, N=8), vehicle-treated/endotoxemic rats (closed bars, N=8), leptin-treated/endotoxemic rats (grey bars, N=8), and leptin-treated/saline-treated rats (dark grey bars, N=8). Oxidative stress was measured as the normalized DHE fluorescence changes. Values are expressed as the mean ± SD. *, $p<0.05$, assessed by one-way ANOVA (Kruskal-Wallis) and Dunn's post-test.

Supplementary Figure S1

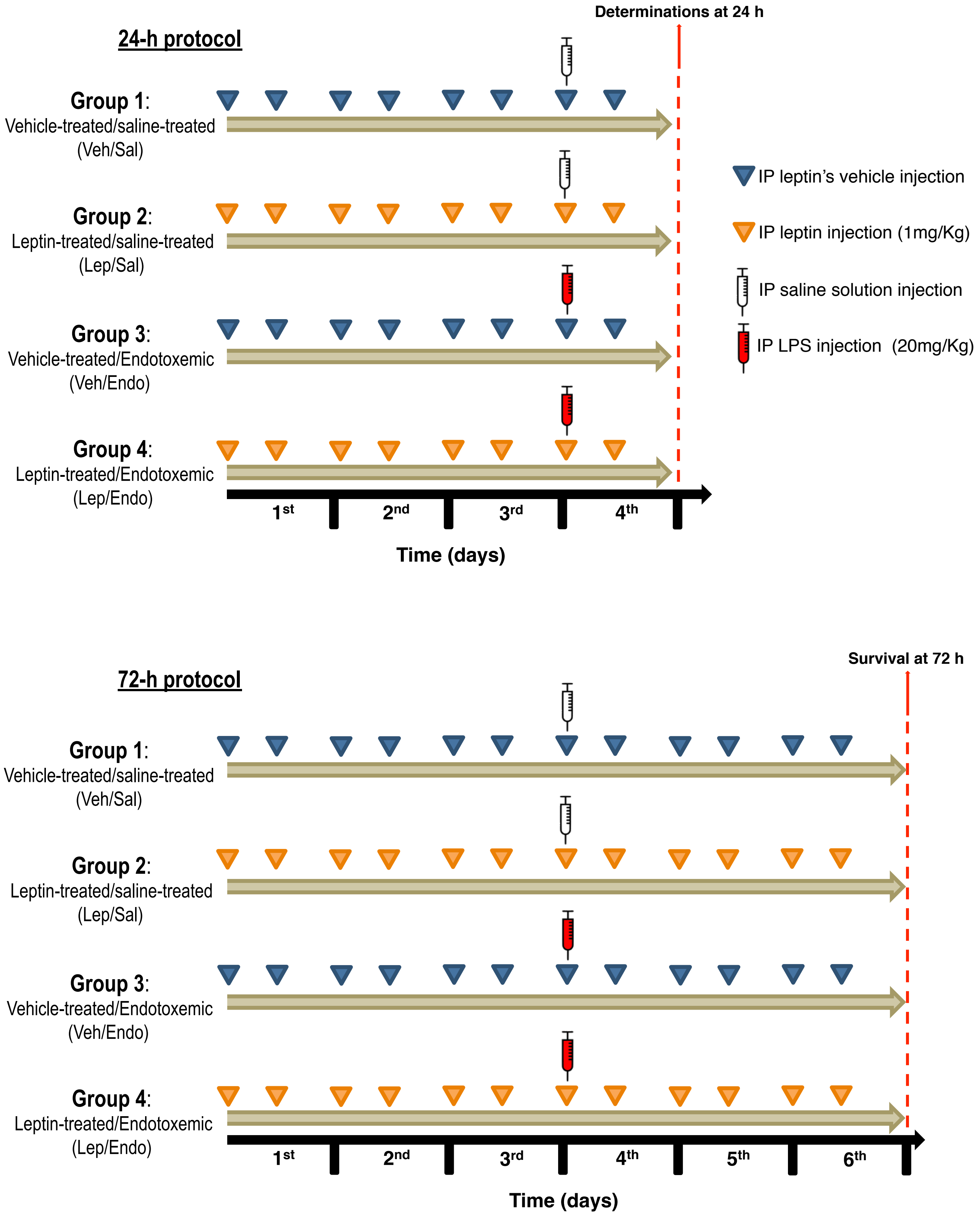

Supplementary Figure S2

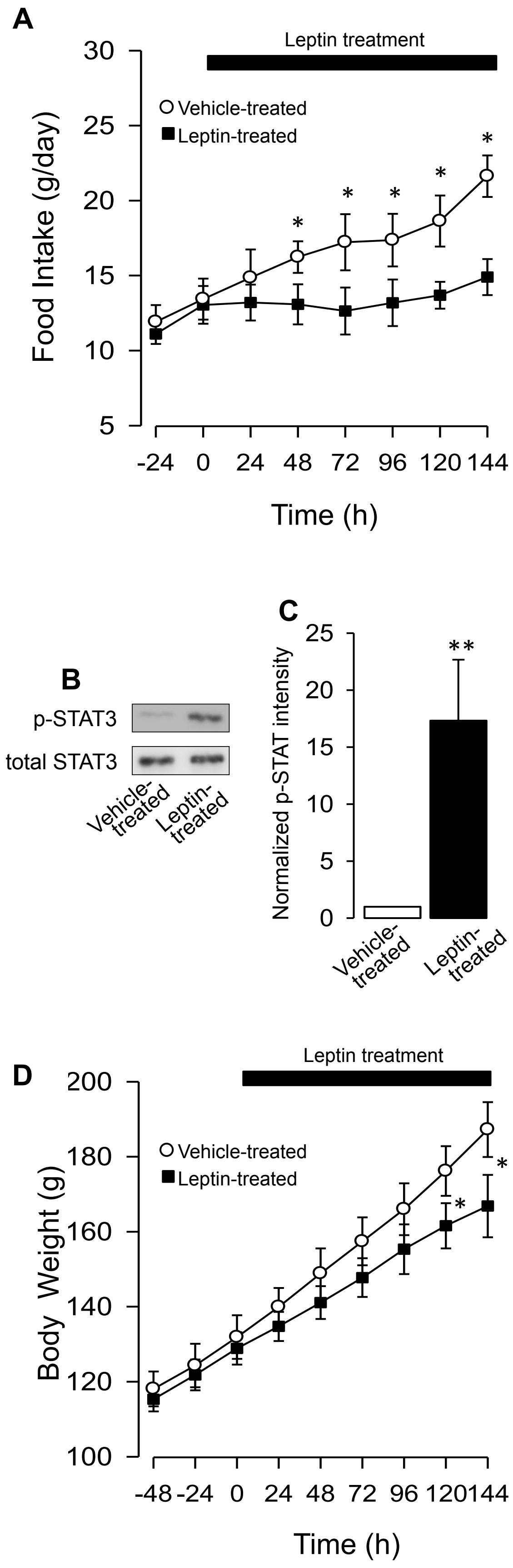

Supplementary Figure S3

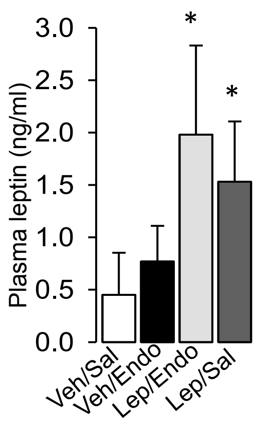

Supplementary Figure S4

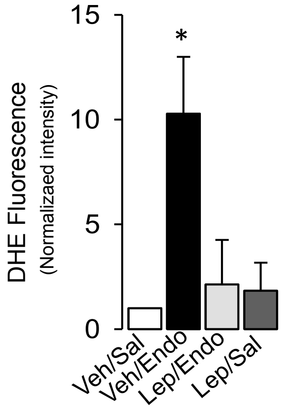

Supplement: Supplementary file 1 [file Data_Sheet_1.PDF]
